# Cryogenic cave carbonate and implications for thawing permafrost at Winter Wonderland Cave, Utah, USA

**DOI:** 10.1038/s41598-021-85658-9

**Published:** 2021-03-19

**Authors:** Jeffrey Munroe, Kristin Kimble, Christoph Spötl, Gabriela Serrato Marks, David McGee, David Herron

**Affiliations:** 1 Geology Department, Middlebury College, Middlebury, VT 05753 USA; 2 Institute of Geology, University of Innsbruck, 6020 Innsbruck, Austria; 3 Department of Earth, Atmospheric, and Planetary Sciences, Massachusetts Institute of Technology, Cambridge, MA 02142 USA; 4 USDA-Forest Service, Ashley National Forest, Duchesne, UT 84021 USA

**Keywords:** Cryospheric science, Palaeoclimate

## Abstract

Winter Wonderland Cave contains perennial ice associated with two types of cryogenic cave carbonate (CCC) formed during the freezing of water. CCC_fine_ is characterized by relatively high δ^13^C values, whereas CCC_coarse_ exhibits notably low δ^18^O values indicating precipitation under (semi)closed-system conditions in a pool of residual water beneath an ice lid. Previous work has concluded that CCC_coarse_ forms during permafrost thaw, making the presence of this precipitate a valuable indicator of past cryospheric change. Available geochronologic evidence indicates that CCC formation in this cave is a Late Holocene or contemporary process, and field observations suggest that the cave thermal regime recently changed in a manner that permits the ingress of liquid water. This is the first documented occurence of CCC_coarse_ in the Western Hemisphere and one of only a few locations where these minerals have been found in association with ice. Winter Wonderland Cave is a natural laboratory for studying CCC genesis.

## Introduction

The cryosphere is responding rapidly to climate warming, and paleoclimate records are critical for understanding the novelty of these responses^1,2^. A persistent challenge is that many paleoclimate archives are biased toward extremes, for instance the most extensive glacial advances^3^, most widespread periglacial conditions^4^, or sea level high- and low-stands^5^. Yet records of past climatic transition are crucial for placing contemporary global change into a longer-term context^6^. This is especially true in Arctic and high mountain environments where temperatures are warming rapidly^7,8^, leading to dramatic diminishment of ice extent^9,10^ and degradation of permafrost^11–13^. Thus there is an urgent need for better records of how the cryosphere changed in the past, to clarify when and how rapidly changes occurred, and in what pattern these changes unfolded across the landscape.

A unique proxy demonstrated to provide information about past episodes of permafrost thaw, a transition that is often particularly disruptive to landscapes, ecosystems and infrastructure, is cryogenic cave carbonate (CCC). These minerals form when liquid water enters a cave containing subzero temperature conditions^14^. As this water freezes, solutes are concentrated in the residual liquid until saturation is reached and precipitation of CCC is induced^15,16^. Although seasonal subzero conditions can be created inside a cave entrance by winter cold, more significant permanently subzero conditions are maintained in so-called “ice caves” by ventilation regimes that preferentially allow the ingress of winter air while excluding summer warmth^17,18^. For instance, caves with a single downward sloping entrance can trap cold air through density settling in winter; air that is not replaced by warmer, less dense air in the summer. Alternatively, caves with multiple entrances at different elevations are susceptible to chimney effects that support freezing conditions where large amounts of cold air are pulled into the cave in winter^17,19^. Either way, CCC is precipitated when water containing sufficient dissolved solutes encounters the subzero conditions.

Two types of CCC have been identified. One group, referred to as CCC_fine_, forms when a film of water freezes on the surface of pre-existing ice^20^. The large surface area of this thin layer facilitates degassing of CO_2_, preferentially removing ^12^C, and leaving the remaining water enriched in ^13^C through disequilibrium effects^21,22^. Simultaneously, the preferential incorporation of ^18^O in the ice is counterbalanced by evaporation of lighter ^16^O from the water surface, yielding a characteristically higher δ^13^C and relatively unaltered δ^18^O values^20^. The ice involved in the formation of CCC_fine_ does not need to be perennial, however the presence of CCC_fine_ in a currently ice-free cave is nonetheless clear evidence for colder conditions in the past.

In contrast, CCC_coarse_ is a more specific proxy because it forms when a migrating permafrost table driven by thaw intersects the upper part of a cave, allowing dripwater to enter and form pools on the ice surface^23,24^. Because the temperature in the main part of the cave (below the permafrost table) is still subzero, these pools freeze over, creating (semi)closed-system conditions in which degassing of CO_2_ and evaporation of water are greatly reduced^23^. Slow freezing of these isolated volumes of water precipitates CCC_coarse_ distinguished by notably lower values of δ^18^O compared with CCC_fine_^14,16,25^. As is apparent throughout Arctic and high mountain environments today, elevation of ground temperature above 0 °C, thawing of permafrost, and melting of ground ice are step-changes with dramatic repercussions for landscape and environmental evolution^26^. CCC_coarse_, therefore, is a critical proxy because it records past episodes of permafrost thaw.

Here we report the first discovery of CCC_coarse_ in North America from an unusual setting where apparently young CCC is present in association with modern perennial ice. Our work provides an important point of comparison for CCC from well-studied caves in Europe, and documents a setting in which theories for CCC genesis could be evaluated.

### Location

**Figure 1:**
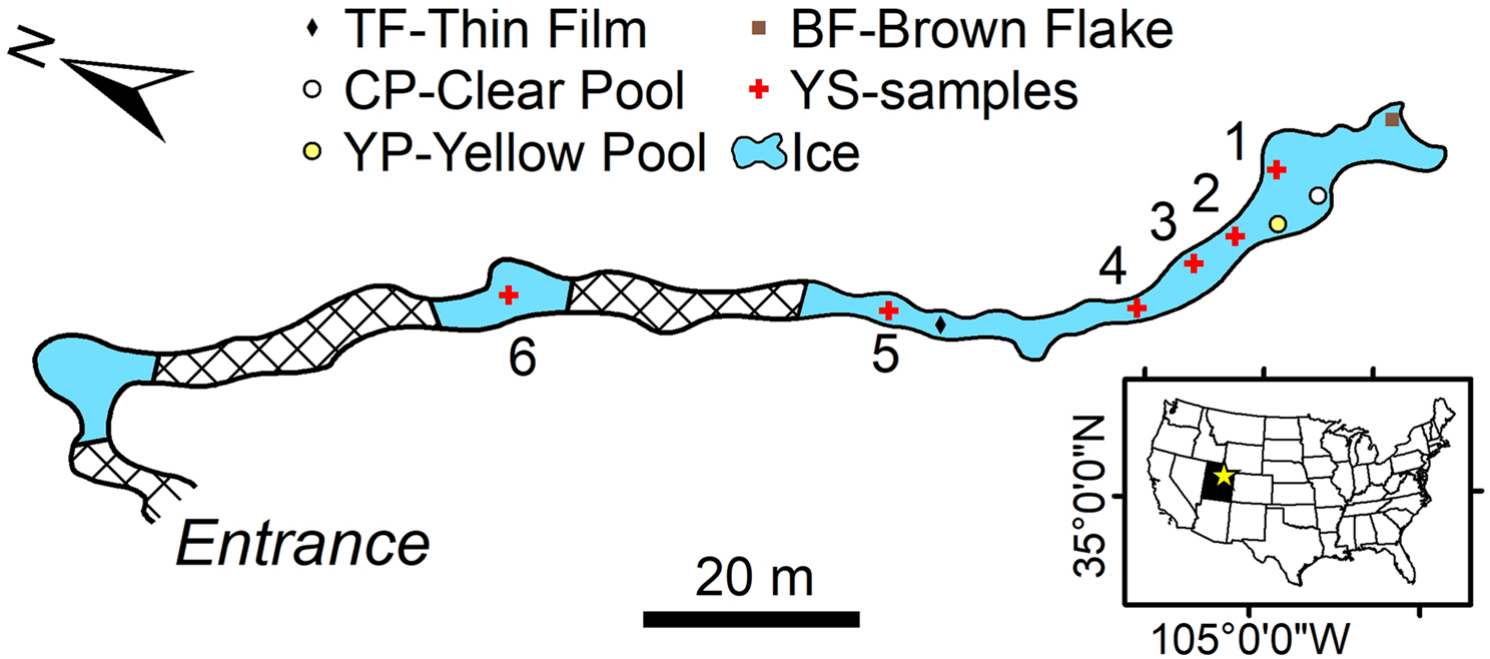
Map of Winter Wonderland Cave showing sections of the cave floored by ice (blue), breakdown (hatched pattern), and sampling locations. Inset shows the location of the cave in northeastern Utah (star).

**Figure 2:**
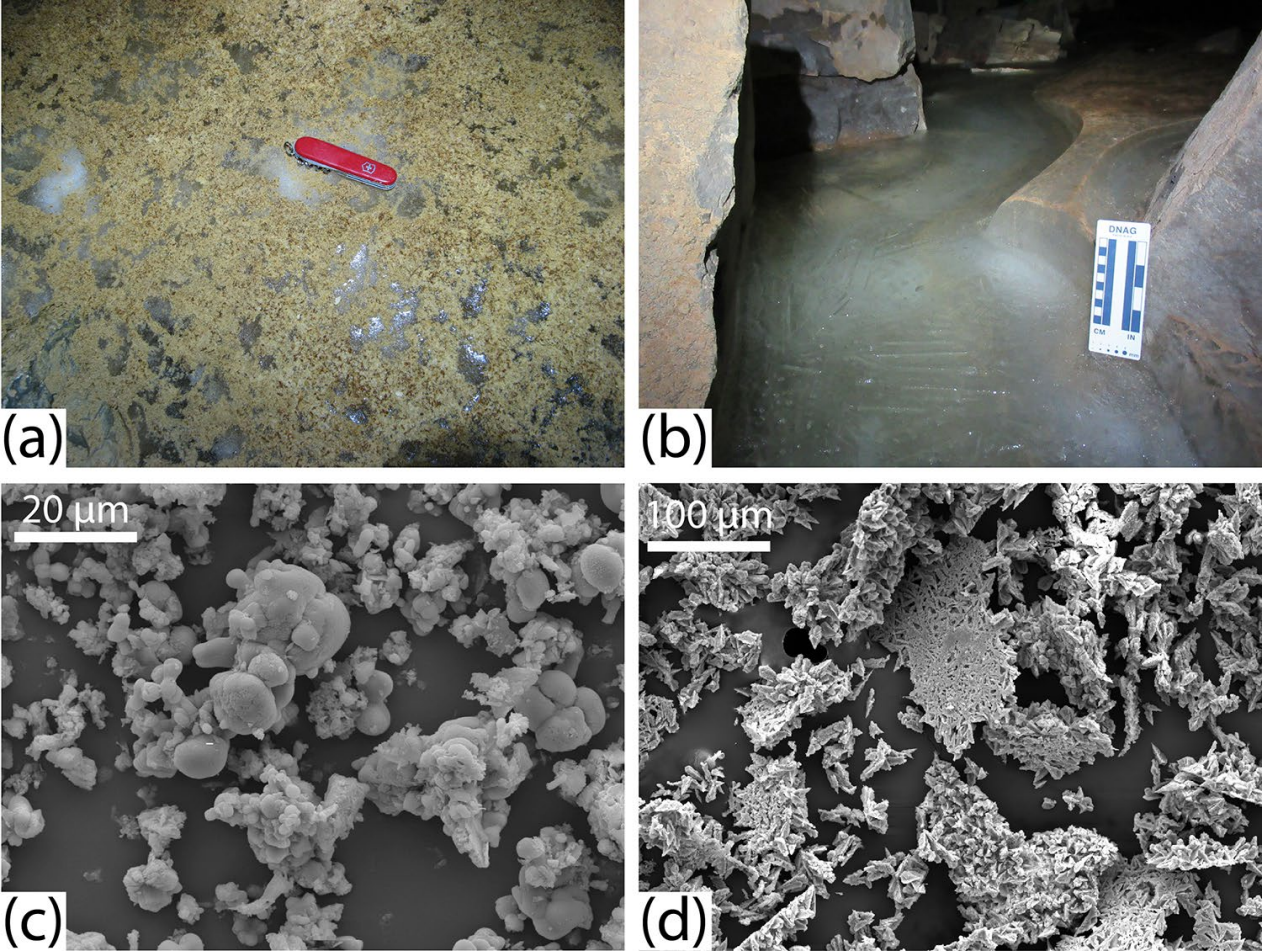
(**a**) Loose CCC_coarse_ on the surface of the ice. Red knife is 10 cm long. (**b**) Pool of water with a lid of ice along the edge of the perennial ice. (**c**) SEM image of sample YS-6 (CCC_coarse_) showing calcite spherules. (**d**) SEM image of sample TF (CCC_coarse_) showing broken calcite rafts.

The CCC studied in this project was collected from Winter Wonderland Cave (WWC) in the Uinta Mountains of northeastern Utah, USA (Fig. 1). This solution cave has developed in the Carboniferous-age Madison Limestone, a regionally extensive rock unit that in this area consists of fine to coarse-grained dolomite and limestone, with locally abundant nodules of chert^27^. The entrance to WWC is at an elevation of 3140 m asl in a north-facing cliff at the edge of the karstified Blind Stream Plateau (40.53°N, 110.73°W), a sparsely forested subalpine landscape. The mean annual air temperature (1985–2020) at the Brown Duck snowpack telemetry (SNOTEL) station at a similar elevation (3223 m) 12 km to the east of WWC is 0.3 °C (https://wcc.sc.egov.usda.gov/nwcc/site?sitenum=368). Elsewhere in the Uinta Mountains, the Chepeta remote automated weather station (RAWS) at an elevation of 3694 m recorded a mean temperature of − 2.0 °C between 2000 and 2019 (https://wrcc.dri.edu/cgi-bin/rawMAIN.pl?utCHEP). Inside WWC temperatures were consistently subzero from 2016 to 2018 (with a mean of − 0.5 °C) because of a ventilation regime that brings cold winter air in through the main entrance, which is elevated above the main part of the cave, and inhibits the entry of warm air in the summer. As a result, about half of the cave, which has a mapped length of 245 m, is floored by perennial ice up to 3 m thick. CCC is present as a lag on the ice surface, drapes rocks emerging from the sublimating ice, and occurs as discrete layers within the ice body (Fig. 2a). Additional details about the cave, its climatology, and the perennial ice are presented in Munroe (2021).

Representative CCC samples, numbered YS-1 through YS-6 (Table S1) were collected from the ice surface along with a sample (BF) from within an exposed stratigraphic section through the ice, and three samples that were submerged in small (~ 10–30 cm deep) pools (Fig. 2b) of water filling depressions in the ice (CP, YP, and TF). Additional samples of Madison Limestone bedrock and water from pools were also collected for analysis.

## Results and interpretation

### CCC morphology

An array of morphologies is present in the CCC samples from WWC (Figs. 2 and S1). The smallest size fractions are spherulitic, with larger aggregates reaching diameters of 50 µm, and individual spherules from 10 to 20 µm in diameter (Figs. 2c and S1a,b). This observation is corroborated by results from laser scattering, which reveal mean grain sizes from 24 to 42 µm in the < 75-µm fraction. Rounded grains are typically clumped in botryoidal aggregates with smooth surfaces, and dumbbell structures are common. Occasionally more sharp-edged morphologies are present, with needle-like points that rarely exceed 5 μm in length. Larger size fractions (75–250 μm) contain rafts of aggregated grains in excess of 200 μm long. The TF samples, which were collected from the water surface, are characterized by arrow-shaped blades 10–20 µm long, grouped into thin rafts (Figs. 2d and S1c). All of these observations match CCC morphologies reported in the literature. For instance, previous work has established that CCC often occurs as raft-like aggregates of crystals, and spherical forms^23,24,28^. Rafts are generally flat and composed of interlocking crystals in a manner similar to floating carbonate minerals reported from non-cryogenic cave environments^29^. Crystal splitting has been invoked as a mechanism for the growth of spherical forms^30^, which are sometimes superimposed on a branching, sheaf-life skeleton^31^. Finally, spherules are reported to display smooth surfaces, and often connect during growth to form dumbbell shapes^23^ or chains^24^.

On the other hand, the CCC in WWC is considerably finer than precipitates with similarly low δ^18^O values reported from European caves. Although there is a wide range in sizes, CCC is typically described as having crystals from < 1 to ~ 40 mm^23,24,28^ compared with the 10–20-µm diameter spherules common in WWC. The significance of this difference is unclear; it may simply indicate that CCC in WWC formed from smaller-volume pools, or from water with a lower total dissolved load.

### Types of CCC present in WWC

**Figure 3:**
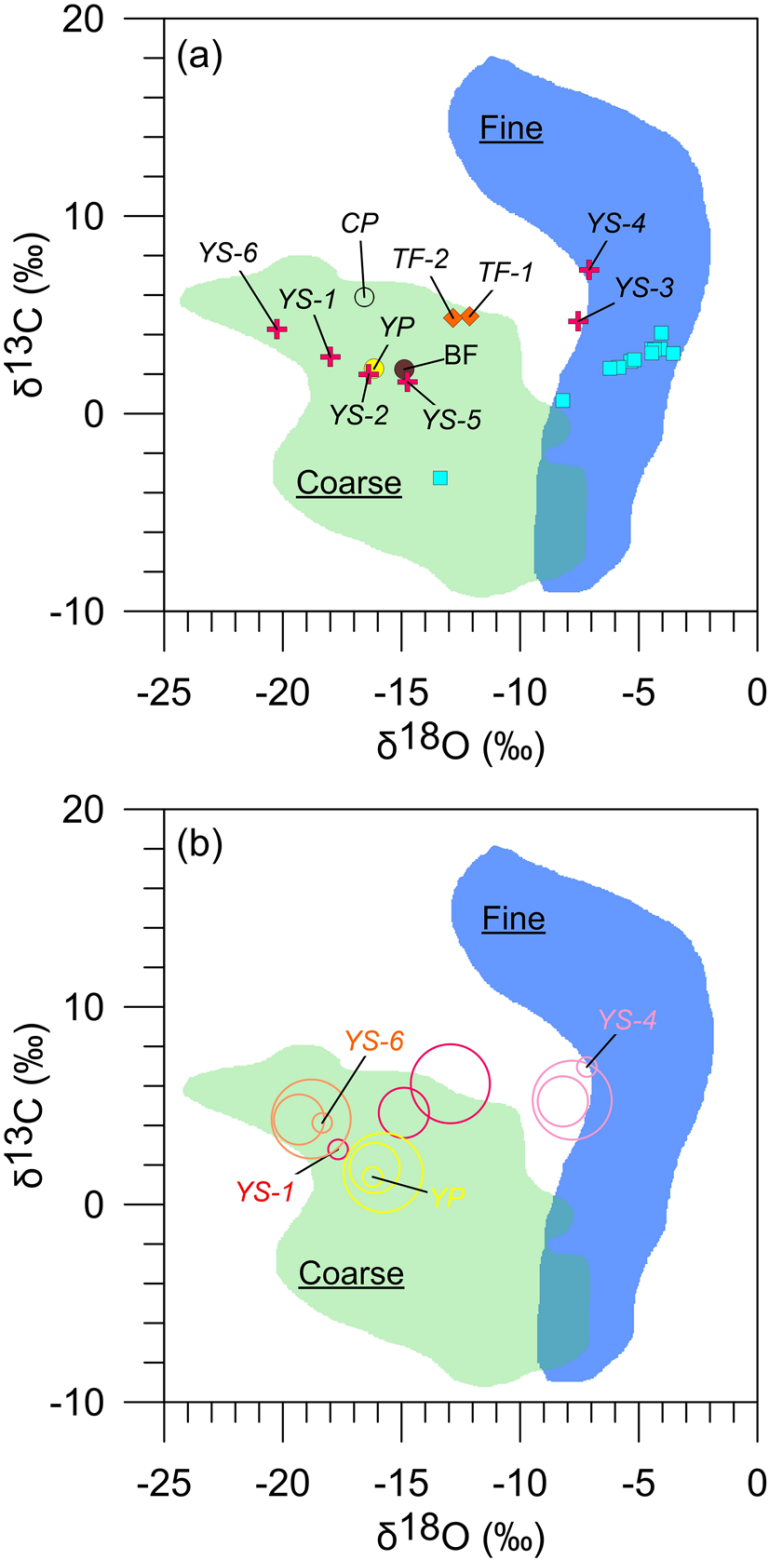
(**a**) Isotope data for samples from WWC: blue squares are bedrock; red crosses are CCC from the ice surface; CP and YP are CCC from the “clear” and the “yellow” pools, respectively; BF is from a layer of CCC within the perennial ice body. The blue and green fields delineate the typical range of isotope values for CCC_fine_ and CCC_coarse_ after^28^. (**b**) Isotope values for samples YS-1, YS-4, YS-6, and YP presented by size fraction: large circle, 165–250 µm; medium circle, 75–165 µm; small circle, < 75 µm.

Although, as their names suggest, early studies differentiated CCC_fine_ and CCC_coarse_ by grain size, δ^13^C and δ^18^O values reflecting different C and O isotope fractionation mechanisms are now recognized as the only valid criteria for distinguishing between the two groups^20^. With this in mind, values of δ^13^C and δ^18^O indicate that both types of CCC are present in WWC (Fig. 3a), despite no consistent distinction in their grain size distributions. Samples YS-3 and YS-4 have δ^13^C and δ^18^O values averaging 6‰ and − 7.3‰ respectively (Table S2), suggesting that both are CCC_fine_. In contrast, samples YS-1, YS-2, YS-5, YS-6, BF, CP, and YP have lower values of δ^13^C, averaging ~ 3.0‰, and notably lower δ^18^O values, averaging − 16.5‰. These values identify these samples as CCC_coarse_ (Table S2). Sample TF also plots in the CCC_coarse_ field (Fig. 3a), although it was collected from a pool that had just begun to freeze, which may explain why its δ^18^O value is less negative than the other CCC_coarse_ samples. For comparison, samples of the Madison Limestone collected in WWC have an average δ^13^C value of 2.2‰ and δ^18^O value of − 5.9‰ (Fig. 3a). Consistency of stable isotope values within individual size fractions (< 75 µm, 75–165 µm, and 165–250 µm) emphasizes that YS-5, YS-6, CP, and YP are pure CCC_coarse_ (Fig. 3b). In YS-1 and YS-2, CCC_coarse_ is concentrated in the < 75-µm fraction. In contrast, YS-4 is consistently CCC_fine_ in all size fractions.

The results of additional analyses permit further interpretation of these samples. XRD reveals that YS-3 and YS-4 (CCC_fine_) are a mixture of calcite and quartz (Fig. S2), and rounded quartz grains were visible in the SEM images (Fig. S1d). The presence of quartz is confirmed in the XRF results, where sample YS-4 contains 52% SiO_2_ (Table S3)_._ YS-3 and YS-4 also have much higher chondrite-normalized REE values (Table S4, Fig. S3). These observations, along with the isotope data for size fractions presented above, are evidence for a detrital component in these CCC_fine_ samples, which were collected from bedrock shelves 50–100 cm above the current ice surface. In contrast, XRD analysis indicates that the CCC_coarse_ samples from the ice surface are composed solely of calcite, as are the bedrock samples (Fig. S2). Major element analysis (Table S3) reinforces the abundance of calcite in YS-6 (70% as CaO), and Mg is considerably more abundant in YS-6 (12.2% as MgO) than in YS-4 (1.8%). Collectively these analyses support the conclusion that the CCC_coarse_ samples are (nearly) pure precipitates, whereas the CCC_fine_ samples are a mixture of precipitated calcite and detrital minerals.

### CCC ages

Previous studies have successfully applied ^230^Th/U disequilibrium dating^28^ to CCC^16^. Accordingly, an attempt was made to date CCC_coarse_ from WWC (Table S5). The resulting ages are imprecise due to low ^230^Th/^232^Th ratios, a situation that has complicated other work^24,28^, meaning that the correction for detrital ^230^Th, and the uncertainty on that correction, are large. On the other hand, one of the samples analyzed (TF) is clearly modern because it was collected in 2018 from a pool of water that was not present in 2016. The initial ^230^Th/^232^Th for this sample was, therefore, applied to the others (with a 25% 2-σ uncertainty) to refine the age calculations. Results indicate that the CCC in WWC likely formed during the Holocene, and nearly all of the ages have error estimates that overlap with modern (Table S5, Fig. S4). Thus these ages, imprecise as they are, are consistent with CCC formation as a recent or current process in WWC. This result is significant because the majority of published CCC ages are from the Late Pleistocene e.g.^23,32^, with only a few reports of Holocene ages^24,28,33^.

Two additional lines of evidence support the interpretation that the CCC in WWC is young. First, rafts of calcite from sample YS-6 yielded a radiocarbon result of f_Modern_ 1.111 ± 0.005, consistent with formation in the late 20th Century when calibrated with the NH1 bomb curve in OxCal 4.4^34^ (Fig. S5). Second, samples of rodent fecal pellets from the ice beneath the surface lag of CCC yielded radiocarbon ages that calibrate to between AD 1560 and 1830^35^ (median values) using the IntCal20 calibration curve^36^. Together this evidence strongly supports the conclusion that CCC formation in WWC occurred in the late Holocene, and is still occurring today.

### Winter Wonderland Cave as a unique natural laboratory for the study of CCC

Because of its utility as a paleoclimate proxy, numerous studies have investigated CCC e.g.^23,32,37,38^. However, there are only a few reports of CCC from locations outside Eurasia^21,39^, and all of these are categorized isotopically as CCC_fine_. Winter Wonderland Cave is, therefore, the first location in the Western Hemisphere where the unique paleoclimate indicator CCC_coarse_ has been identified.

Furthermore, nearly all previous observations of CCC describe these minerals from locations where they are present as loose concentrations of mineral grains on an ice-free cave floor^23,28^. Only twice has CCC_coarse_ been reported in association with modern, perennial ice^33,38^, and a recent comprehensive review noted that *“Despite increasing evidence for Holocene CCC*_*coarse*_*, actively forming sites have not yet been observed”*^14^. In WWC, CCC_coarse_ with late Holocene to modern ages is present in association with perennial ice, making this cave an exceptional natural laboratory in which to study these precipitates and their genesis.

**Figure 4:**
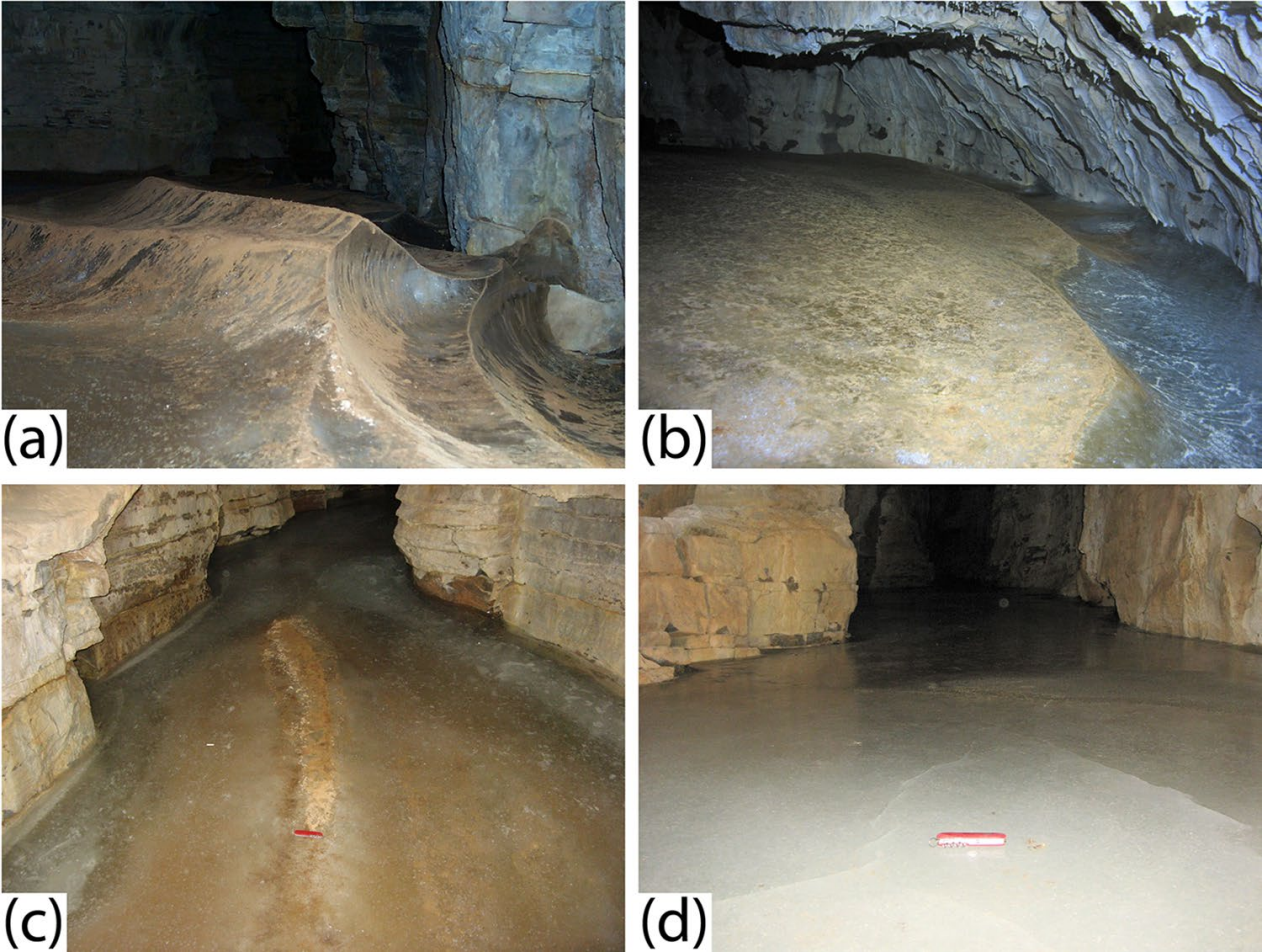
A selection of photographs highlighting recent changes observed in WWC. (**a**) The sculpted surface of the ice in 2016 illustrating intersecting cuspate forms produced by sublimating air currents. The total relief is ~ 30 cm. CCC is visible as tan material on the ice surface. (**b**) A view showing the edge of the ice in 2016 where a ~ 20-cm-deep moat had formed at the contact with the rock wall of the cave. (**c**) The surface of the ice in 2018 illustrating how inflowing water had submerged nearly all of the former sculpted surface beneath new ice. The red knife is located at the end of a ~ 1-m long section of a former crest in the ice that reaches barely above the new ice surface. (**d**) Another view from 2018 where new ice formed from inflowing water has completely inundated the former sculpted ice surface. Red knife is 10 cm long.

Furthermore, firsthand observations indicate that the situation within WWC is consistent with the model for the formation of CCC_coarse_. During summer visits in 2014, 2015, and 2016, the cave was dry and the ice surface exhibited a complex pattern of elongated ridges and troughs with local relief on the order of 30 cm (Fig. 4a-c) formed as a result of sublimation^40^. Short-term studies in an ice cave in Alberta, Canada suggest ice sublimation rates on the order of 3 mm per year^41^, although rates up to 10× higher have also been reported^14^. Either way, extrapolation from these benchmarks suggests that the relief observed on the ice surface in 2016 reflects 10^1^ to 10^2^ years of sublimation without the addition of new water. In contrast, in summer 2018, and again in 2019, liquid water entered the cave, filling many of the furrows, and freezing to create a new ice surface (Fig. 4d). Deeper pools of water had lids of ice from 1 to 5 cm thick (Fig. 2b), precisely the mechanism proposed for the formation of CCC_coarse_^16,23,25^. This water ranged from clear to a deep yellow color, and CCC was observed on the floor of each pool (Table S1). From the available data we cannot determine if this CCC precipitated from the water, or whether it was present as a lag on the ice surface before the pools formed. However, the concentration of solutes in this pool water was very high, with the highest values (K, Mg, and Ca > 200 mg/L) corresponding to the yellow color (Fig. S6), consistent with conditions necessary for mineral precipitation through freezing-induced saturation^15^.

The change from 2016 to 2018 suggests that the thermal state of the epikarst has shifted, at least locally, to allow liquid water to penetrate to the level of the cave (~ 100 m below the ground surface), or to produce meltwater from ice farther back in inaccessible parts of the cave system. Under extensive permafrost conditions, caves are unlikely to contain ice sourced from dripwater because the permafrost inhibits the downward movement of liquid water^42^. Only when permafrost is degrading is it possible for liquid water to reach a cave where temperatures remain consistently subzero. The available evidence supports the interpretation that WWC and its surrounding bedrock comprise a sporadic permafrost body that is currently undergoing this transition. Given the presence of several meters of layered ice and associated CCC in WWC, it seems likely that this site has had a complicated thermal history, during which lengthy (decadal scale or longer) intervals of permafrost condition in the epikarst were interrupted by thawing events that allowed water to enter the cave and create new layers of ice. The influx of water to the cave observed in the past two summers is, therefore, not entirely unprecedented. Nonetheless, numerical modeling suggests that the time window between the onset of permafrost thaw in the epikarst and ultimate loss of cave ice deposits can be relatively short^43^, and studies have noted that cave ice is rapidly ablating in various locations around the world^44^. Future investigations in WWC should take advantage of this singular opportunity to observe the formation of different types of CCC in (near) real time, with the goal of improving our ability to use the presence of these features in currently ice-free caves as a dateable indicator of past permafrost thaw^23^.

## Conclusion

Winter Wonderland Cave in the Uinta Mountains of Utah contains cryogenic cave carbonate (CCC) associated with perennial ice. Two types of CCC with different genesis are present and can be distinguished on the basis of O and C isotope values. CCC_fine_ is produced through open system freezing as a thin film of water flows over the ice surface. In contrast, CCC_coarse_ is produced by (semi) closed-system freezing in deeper pools of water beneath thickening lids of ice. These conditions arise during permafrost thaw, thus CCC_coarse_ records past episodes of permafrost degradation. Available age control suggests that CCC formation occurred in this cave during the late Holocene and may be a contemporary process. This cave is the first location in the world where such young CCC_coarse_ has been found, and is the first cave in the Western Hemisphere where CCC_coarse_ has been identified. Records of past cryosphere transitions are critical context for assessing contemporary changes in Arctic and Alpine environments. Winter Wonderland Cave provides a singular opportunity to test and improve theories of CCC genesis that will ultimately allow better insight into how permafrost responded to past climatic transitions.

## Methods

### Sample preparation and analysis

At Middlebury College, CCC samples were wet-sieved into < 75, 75–250, and > 250-µm size fractions. Each fraction was placed in a 50-mL centrifuge tube, and after fine sediment had settled, excess water was carefully decanted. Samples were then frozen in a − 40 °C freezer for 4 h. The uncapped centrifuge tubes were then dried overnight in a Labconco FreeZone 6 L freeze dryer. The resulting powder was lightly ground with a clean mortar and pestle and returned to each tube.

### Scanning electron microscopy

A Tescan Vega 3 LMU scanning electron microscope (SEM) at Middlebury College was used to acquire images of the CCC in different grain size fractions. Samples were adhered to metal disks with double sided tape, coated in gold palladium, and images were captured at different magnifications in order to compare CCC morphologies. Energy dispersive X-ray spectroscopy (EDS) was applied to samples YS-3, YS-6, TS-2, CP, YP, and TF after carbon coating to determine if varying morphologies correspond to different elemental compositions. The semi-quantitative elemental results were converted to weight % oxides to differentiate between calcite, dolomite, and quartz. AZtecOne software was used to capture secondary electron and backscatter images and for elemental analysis. Weight % oxide data were transformed into structural formulae for common minerals.

### Grain size distribution

The grain size distribution of the < 75-µm fraction of each sample was determined using a Horiba LA-950. In preparation for analysis, samples were dispersed in distilled water, mixed for 30 s using a Vortex-T Genie 2, and sonified with an FS20D sonifying water bath for 1 min. A clean syringe was used to transfer an aliquot of each sample to the Horiba. Sample runs were duplicated to assess consistency. Grain size distribution data were presented as volume percentages of sand (coarse, medium, and fine), silt (coarse, medium, fine, and very fine), clay (2–1 µm), and colloid (< 1 µm).

### X-ray diffraction

Samples of the 75–250-µm fraction, as well as rock samples powdered in a shatterbox, were analyzed on a Bruker D8 Advance Model X-Ray Diffractometer at Middlebury College. The mineralogy of several samples was compared across different size fractions (< 75 µm and 75–250 µm) to detect any compositional differences. As no obvious differences were detected, the fractions < 75 µm and 75–250 µm were used interchangeably for future analyses depending on the volume of sample remaining. Nine samples were analyzed as bulk powders from 2° to 50° 2Θ. Samples CP, YP, and TF were not large enough for a bulk powder analysis, so they were transferred to a glass slide and analyzed as oriented powders, and scanned from 2°–40° 2Θ. The diffractometer operated at 40 kV and 40 mA with a solid state detector, theta-theta goniometer, and CuKα radiation. Mineral abundances were assessed using intensity ratios, and known mineral spectra peaks were identified by comparison with standard reference patterns.

### X-ray fluorescence

Samples of the 75–250-µm size fraction were analyzed for major element composition using X-ray fluorescence (XRF) at Middlebury College. Samples were ignited at 1000 °C for 30 min in a Leco TGA-701 thermogravimetric analyzer prior to analysis to remove organics and interstitial water. Ignited samples were ground to a fine powder and combined in a 10:1 ratio of lithium borate fluxing agent to sample (6.0 g fluxing agent and 0.6 g of sample). The combined sample and fluxing agent were melted in a Claisse LeNeo fluxer at 1050 °C to form a glass disk. This process was repeated for samples YS-4, YS-6, TS-1, TS-2, and one bedrock sample; other samples did not contain sufficient mass in the desired particle size range for fluxing. Glass disks were analyzed using a Thermo Scientific ARL QuantX energy dispersive (ED) XRF spectrometer to measure major elements. A glass disk made from Standard Reference Material 88B (dolomitic limestone) was included in each run to assess the accuracy of measurements. The analysis quantified the abundance of oxides, which were converted to atomic abundances of major elements (Si, Ti, Al, Fe, Mn, Mg, Ca, Na, K, P).

### Inductively coupled plasma mass spectrometry

Seven samples of the < 75-µm fraction (YS-3, YS-4, YS-5, YS-6, TS-1, TS-2, and a bedrock sample), and an additional YS-4 sample of the 75–250-µm fraction, were prepared for analysis with inductively coupled plasma mass spectrometry (ICP-MS) to determine the abundance of trace and rare earth elements. Dried samples were combined in a 9:1 ratio of lithium metaborate fluxing agent to sample (1.8 g LiBO_2_ and 0.2 g sample). A Claisse LeNeo fluxer was used to melt the sample and fluxing agent and dissolve the melt in 5% HNO_3_. Dissolved CCC and rock samples, along with samples of water from the cave were analyzed with a Thermo Scientific iCAP Q ICP-MS at Middlebury College in conjunction with Qtegra ISDS software. Calibration curves were generated for each run, and quality control with internal standards was used to drift correct results after every five samples. Standard Reference Material 88B (dolomitic limestone) was analyzed at the beginning and end of every batch of CCC samples, and National Institute of Standards and Technology (NIST) standard 1643f. (trace elements in water) was run in conjunction with water samples.

### C and O stable isotopes

Eleven samples (all of < 75-µm size fraction except for CP and YP where the 75–250-µm fraction was used) were prepared for δ^18^O and δ^13^C stable isotope analysis in the Department of Geology at Union College using a Thermo GasBench II connected to a Thermo Delta Advantage mass spectrometer in continuous flow mode. Analytical uncertainties were better than 0.1 ‰ (1σ) for δ^18^O and 0.05‰ (1σ) for δ^13^C. All values were calibrated against international standards and reported in permil (‰) relative to Vienna Pee Dee belemnite (VPDB).

Eleven samples of bedrock, along with additional wet-sieved size fractions (< 75, 75–165, and 165–250 µm) of samples YS-1 through YS-6, CP, and YP were analyzed in the Institute for Geology at the University of Innsbruck on a Thermo Scientific Delta V Plus isotope ratio mass spectrometer (IRMS) connected to a GasBench II^45^. This system produces a typical precision (1σ) of ± 0.06‰ for δ^13^C and ± 0.08‰ for δ^18^O ref.^46^. All samples were run in duplicate.

### ^230^Th/^234^U disequilibrium dating

Five samples (YS-5, YS-6, CP, YP, and TF) were analyzed using U-Th radiometric dating techniques in the Department of Earth, Atmospheric, and Planetary Sciences at the Massachusetts Institute of Technology. Two samples were divided into three replicates (YS-5 A, B, C and YS-6 A, B, C) to assess reliability of the dating for young samples with a high potential for Th contamination. One replicate for each sample did not produce a result due to a low yield in chemistry. Approximately 0.03 g of each sample was combined with a ^229^Th-^233^U-^236^U spike, then digested and purified via iron coprecipitation and ion exchange chromatography. U and Th were analyzed on separate aliquots using a Nu Plasma II-ES multi-collector ICP-MS equipped with a CETAC Aridus II desolvating nebulizer following previously published protocols^47^. U-Th ages were calculated using standard decay constants for ^230^Th, ^234^U, and ^238^U^48–50^. Ages were recalculated by assigning sample TF an age of -61 years BP (where present is defined as AD 1950), given the observation that it formed in the past few years. Reported errors for ^238^U and ^232^Th concentrations are estimated to be ± 1% (2-sigma) due to uncertainties in spike concentration, whereas analytical errors are smaller. Variability of initial ^230^Th/^232^Th throughout the cave is assumed to be ± 25% at 2-sigma.

## Supplementary information

Supplementary information.

## Data Availability

The dataset generated in this study has been deposited in the Hydroshare repository and will have the doi https://doi.org/10.4211/hs.b5f0000096174af9af94fe62bd2065d6 after publication*.*
