# Supplementary material for: Cryogenic cave carbonate and implications for thawing permafrost at Winter Wonderland Cave, Utah, USA
Source: Sci Rep. 2021 Mar 19;11:6430. doi: 10.1038/s41598-021-85658-9 (PMC7979826; doi:10.1038/s41598-021-85658-9)
Supplement: Supplementary file 1 — Supplementary information. [file 41598_2021_85658_MOESM1_ESM.docx]

**Supplementary Information** for “*Cryogenic Cave Carbonate and Implications for Thawing Permafrost at Winter Wonderland Cave, Utah, USA*”

Jeffrey Munroe^1*^, Kristin Kimble^1^, Christoph Spötl^2^, Gabriela Serrato Marks^3^, David McGee^3^, and David Herron^4^

^1^Geology Department, Middlebury College, Middlebury, VT, 05753, USA.

^2^Institute of Geology, University of Innsbruck, 6020 Innsbruck, Austria.

^3^Department of Earth, Atmospheric, and Planetary Sciences, Massachusetts Institute of Technology, Cambridge, MA, 02142, USA.

^4^USDA-Forest Service, Ashley National Forest, Duchesne, UT, 84021, USA.

*Corresponding author: Jeffrey Munroe (jmunroe@middlebury.edu)

**Table S1. CCC Samples Collected from Winter Wonderland Cave**

| **Sample Name** | **Image** | **Location** | **CCC type and mineralogy** |
| --- | --- | --- | --- |
| YS-1 | 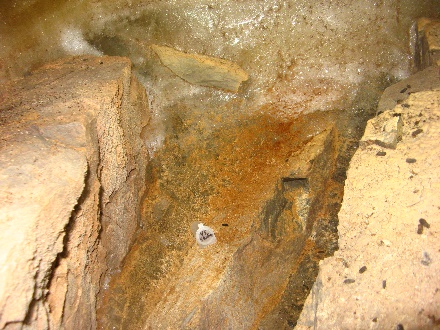 | On ice surface | CCC_coarse_  Calcite |
| YS-2 | 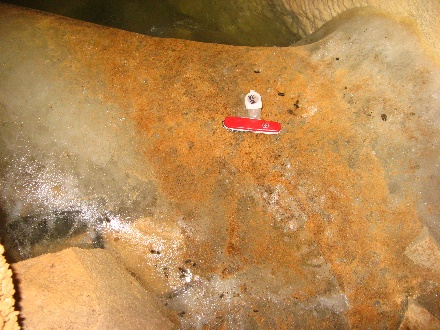 | On ice surface | CCC_coarse_  Calcite |
| YS-3 | 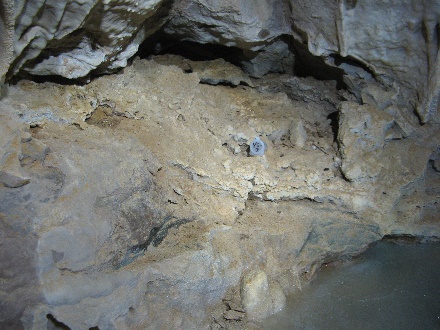 | On ledge above ice surface | CCC_fine_  Calcite, Quartz |
| YS-4 | 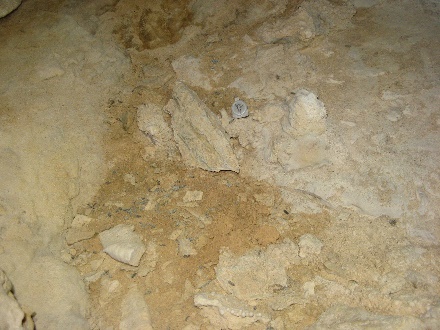 | On ledge above ice surface | CCC_fine_  Calcite, Quartz |
| YS-5 | 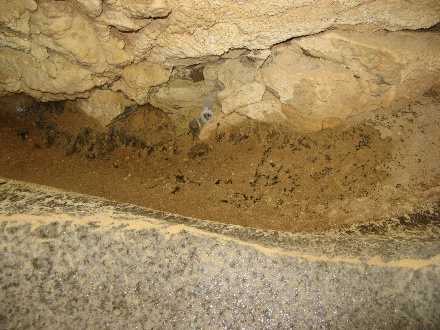 | In moat at base of wall | CCC_coarse_  Calcite |
| YS-6 | 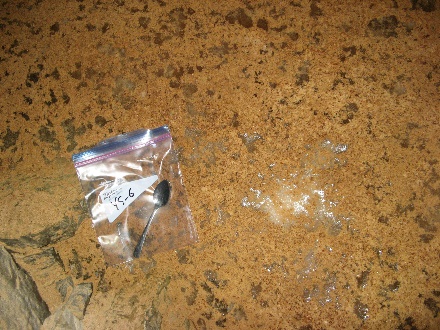 | From ice surface | CCC_coarse_  Calcite |
| CP | 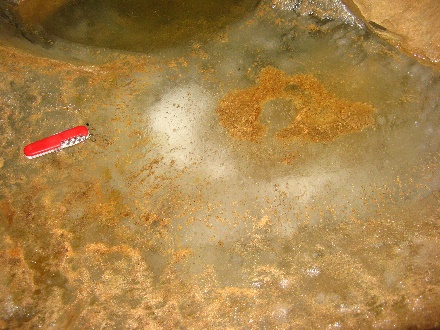 | From clear pool of water on ice surface | CCC_coarse_  Calcite |
| YP | 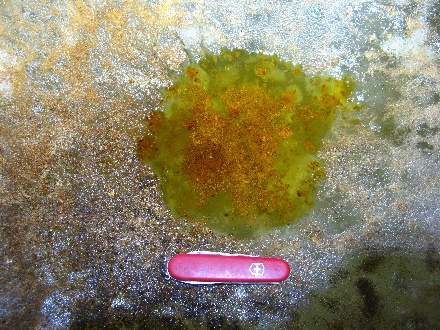 | From yellow pool of water on ice surface | CCC_coarse_  Calcite |
| TF | 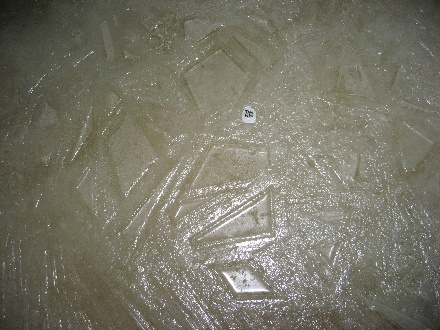 | From surface of water amidst polygonal crystals | CCC_coarse_  Calcite |
| BR | 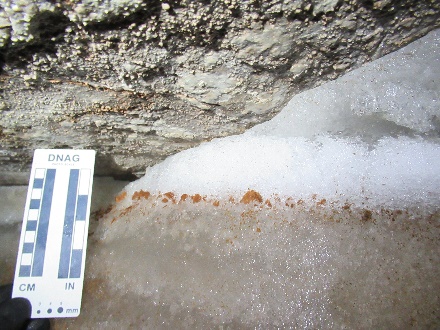 | Collected *in situ* from near top of ice exposure | CCC_coarse_  Calcite |

| **Table 2. Isotope Results from Winter Wonderland Cave** | | | | |  |
| --- | --- | --- | --- | --- | --- |
|  |  |  |  |  |  |
|  |  |  |  |  |  |
| **Sample** | **Size fraction** | **Lab** | **δ^18^O (‰)** | **δ^13^C (‰)** | **Note** |
|  |  |  |  |  |  |
|  |  |  |  |  |  |
| YS-1 | Bulk | Union | -18.00 | 2.87 | CCC_coarse_ |
| YS-2 | Bulk | Union | -16.38 | 2.00 | CCC_coarse_ |
| YS-3 | Bulk | Union | -7.55 | 4.68 | CCC_fine_ |
| YS-4 | Bulk | Union | -7.09 | 7.28 | CCC_fine_ |
| YS-5 | Bulk | Union | -14.75 | 1.61 | CCC_coarse_ |
| YS-6 | Bulk | Union | -20.25 | 4.27 | CCC_coarse_ |
| CP | Bulk | Union | -16.57 | 5.90 | CCC_coarse_ |
| YP | Bulk | Union | -16.16 | 2.27 | CCC_coarse_ |
| TF-1 | Bulk | Union | -12.12 | 4.92 | CCC_coarse_ |
| TF-2 | Bulk | Union | -12.83 | 4.84 | CCC_coarse_ |
| BF - 1a | Bulk | Innsbruck | -14.71 | 2.33 | CCC_coarse_ |
| BF - 1b | Bulk | Innsbruck | -15.06 | 2.16 | CCC_coarse_ |
|  |  |  |  |  |  |
| YS - 1 - 2a | 165-250 | Innsbruck | -12.86 | 6.03 | More CCC_coarse_ in finer fractions |
| YS - 1 - 2b | 165-250 | Innsbruck | -13.03 | 6.20 |  |
| YS - 1 - 3a | 75-165 | Innsbruck | -14.90 | 4.67 |  |
| YS - 1 - 3b | 75-165 | Innsbruck | -14.89 | 4.61 |  |
| YS - 1 - 4a | <75 | Innsbruck | -17.72 | 2.79 |  |
| YS - 1 - 4b | <75 | Innsbruck | -17.63 | 2.80 |  |
| YS - 2 - 2a | 165-250 | Innsbruck | -9.20 | 3.65 | More CCC_coarse_ in finer fractions |
| YS - 2 - 2b | 165-250 | Innsbruck | -9.39 | 3.59 |  |
| YS - 2 - 3a | 75-165 | Innsbruck | -14.43 | 3.05 |  |
| YS - 2 - 3b | 75-165 | Innsbruck | -14.52 | 3.05 |  |
| YS - 2 - 4a | <75 | Innsbruck | -16.18 | 1.66 |  |
| YS - 2 - 4b | <75 | Innsbruck | -16.25 | 1.62 |  |
| YS - 3 | >250 | Innsbruck | -9.48 | 6.76 | CCC_fine_ |
| YS - 3 - 2a | 165-250 | Innsbruck | -14.75 | 2.17 |  |
| YS - 3 - 2b | 165-250 | Innsbruck | -14.60 | 2.15 |  |
| YS - 3 - 3a | 75-165 | Innsbruck | -8.83 | 2.69 |  |
| YS - 3 - 3b | 75-165 | Innsbruck | -- | -- |  |
| YS - 3 - 4a | <75 | Innsbruck | -7.46 | 4.30 |  |
| YS - 3 - 4b | <75 | Innsbruck | -7.27 | 4.45 |  |
| YS-4 | >250 | Innsbruck | -5.05 | 5.95 | CCC_fine_ |
| YS - 4 - 2a | 165-250 | Innsbruck | -7.78 | 5.27 |  |
| YS - 4 - 2b | 165-250 | Innsbruck | -7.84 | 5.30 |  |
| YS - 4 - 3a | 75-165 | Innsbruck | -8.12 | 5.34 |  |
| YS - 4 - 3b | 75-165 | Innsbruck | -8.27 | 5.1 |  |
| YS - 4 - 4a | <75 | Innsbruck | -7.21 | 6.96 |  |
| YS - 4 - 4b | <75 | Innsbruck | -7.17 | 6.95 |  |
| YS - 5 - 2a | 165-250 | Innsbruck | -15.61 | 2.01 | all CCC_coarse_ |
| YS - 5 - 2b | 165-250 | Innsbruck | -15.59 | 2.06 |  |
| YS - 5 - 3a | 75-165 | Innsbruck | -14.57 | 1.82 |  |
| YS - 5 - 3b | 75-165 | Innsbruck | -14.53 | 1.80 |  |
| YS - 5 - 4a | <75 | Innsbruck | -14.37 | 1.33 |  |
| YS - 5 - 4b | <75 | Innsbruck | -14.22 | 1.34 |  |
| YS - 6 - 2a | 165-250 | Innsbruck | -18.60 | 4.31 | all CCC_coarse_ |
| YS - 6 - 2b | 165-250 | Innsbruck | -19.02 | 4.35 |  |
| YS - 6 - 3a | 75-165 | Innsbruck | -19.28 | 4.22 |  |
| YS - 6 - 3b | 75-165 | Innsbruck | -19.35 | 4.39 |  |
| YS - 6 - 4a | <75 | Innsbruck | -18.29 | 4.12 |  |
| YS - 6 - 4b | <75 | Innsbruck | -18.39 | 4.14 |  |
| CP - 2a | 165-250 | Innsbruck | -16.05 | 5.51 | all CCC_coarse_ |
| CP - 2b | 165-250 | Innsbruck | -16.20 | 5.59 |  |
| CP - 3a | 75-165 | Innsbruck | -15.99 | 5.21 |  |
| CP - 3b | 75-165 | Innsbruck | -16.01 | 5.28 |  |
| CP - 4a | <75 | Innsbruck | -15.52 | 4.77 |  |
| CP - 4b | <75 | Innsbruck | -15.66 | 4.72 |  |
| YP - 2a | 165-250 | Innsbruck | -15.90 | 1.62 | all CCC_coarse_ |
| YP - 2b | 165-250 | Innsbruck | -15.65 | 1.62 |  |
| YP - 3a | 75-165 | Innsbruck | -16.09 | 1.91 |  |
| YP - 3b | 75-165 | Innsbruck | -16.17 | 1.79 |  |
| YP - 4a | <75 | Innsbruck | -16.11 | 1.46 |  |
| YP - 4b | <75 | Innsbruck | -16.32 | 1.34 |  |
| BF - 1a | Bulk | Innsbruck | -14.71 | 2.33 |  |
| BF - 1b | Bulk | Innsbruck | -15.06 | 2.16 |  |
| TF - 1a | Bulk | Innsbruck | -12.54 | 4.82 |  |
| TF - 1b | Bulk | Innsbruck | -12.54 | 4.82 |  |
|  |  |  |  |  |  |
| WW - 1a* | Rock | Innsbruck | -5.34 | 2.64 |  |
| WW - 1b | Rock | Innsbruck | -5.19 | 2.73 |  |
| SR-1 | Rock | Innsbruck | -4.45 | 3.26 |  |
| SR-2 | Rock | Innsbruck | -4.09 | 3.28 |  |
| SR-3 | Rock | Innsbruck | -4.34 | 3.24 |  |
| CoD-1 | Rock | Innsbruck | -13.37 | -3.26 | Dolomite |
| CoD-2 | Rock | Innsbruck | -8.20 | 0.66 | Dolomite |
| CoD-3 | Rock | Innsbruck | -3.55 | 3.04 |  |
| FF-1 | Rock | Innsbruck | -5.83 | 2.35 |  |
| FF-3-1 | Rock | Innsbruck | -4.45 | 3.06 |  |
| By-6 | Rock | Innsbruck | -4.04 | 4.08 |  |
| Alcove | Rock | Innsbruck | -6.21 | 2.29 |  |
|  |  |  |  |  |  |
|  |  |  |  |  |  |
| *Rock sample locations include: "WW: near entrance, "SR" near YS-1, "CoD" between entrance | | | | | |
| and YS-6, "FF" between YS-2 and YS-5, "By" near YS-6, and "Alcove" near YS-3 (see Fig. 1) | | | | | |

**
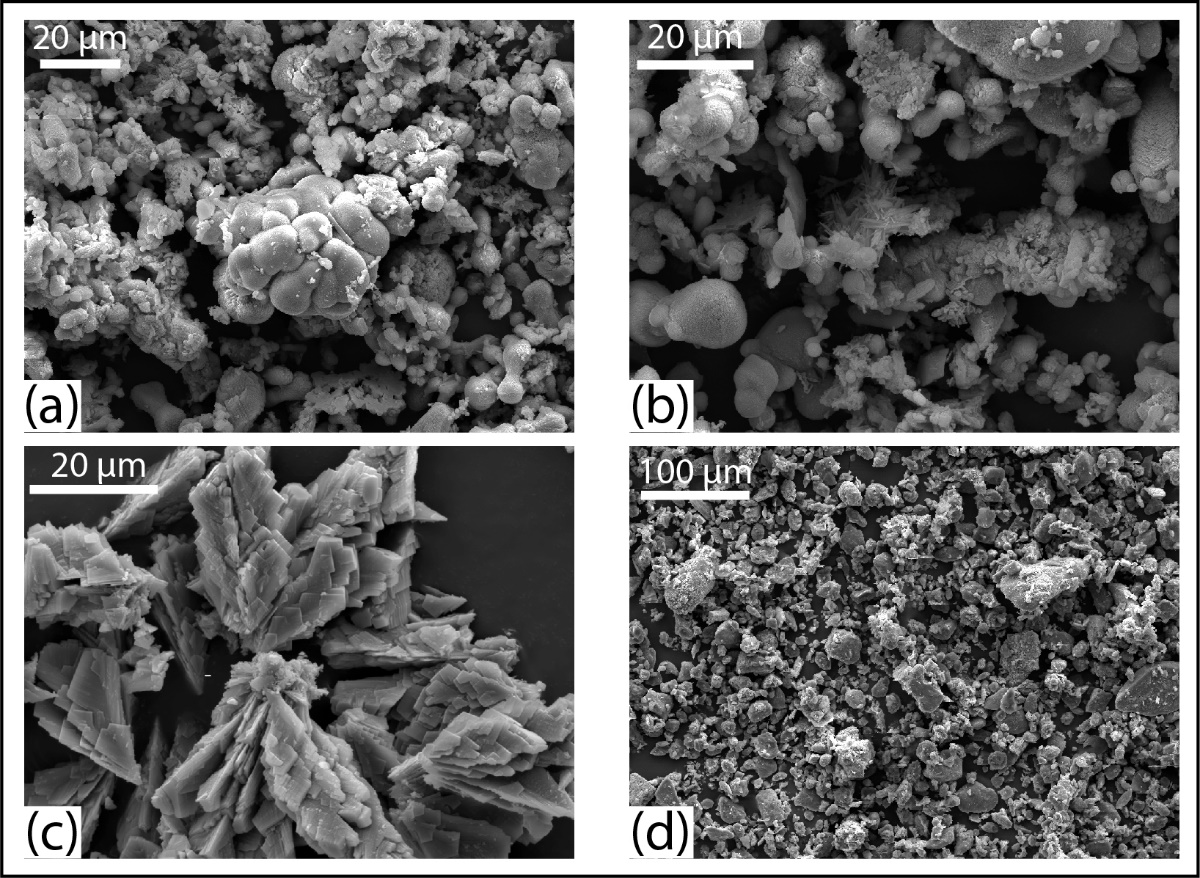
**

**Figure S1**: Additional SEM images of CCC samples from WWC. (a) SEM image of sample YS-1 (CCC_coarse_). (b) SEM image of sample YS-1 (CCC_coarse_). (a) SEM image of sample TF (CCC_coarse_). (a) SEM image of sample YS-3 (CCC_fine_).


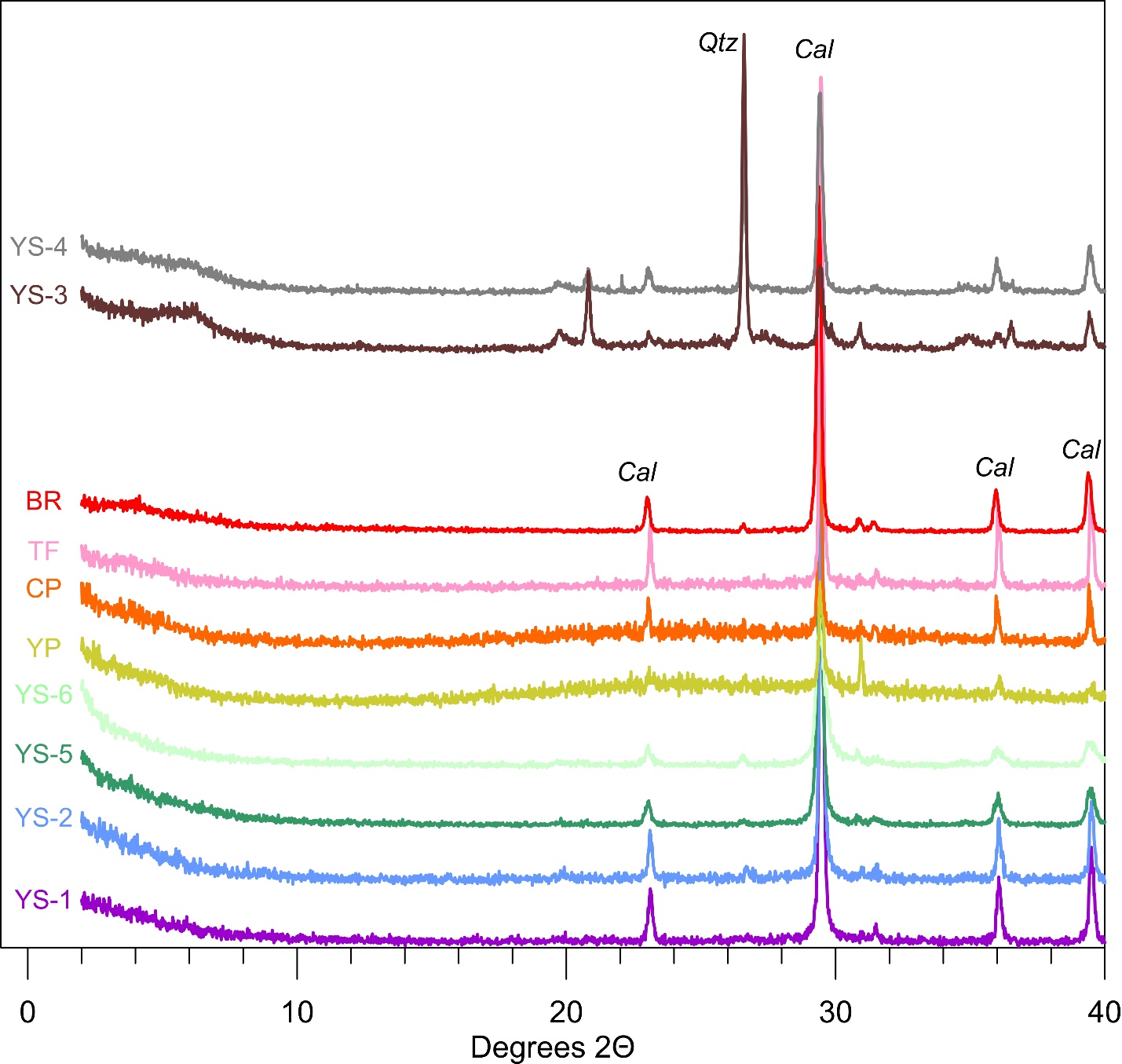


**Figure S2**: XRD patterns for samples from Winter Wonderland Cave. Samples YS-1 through YS-6, YP (Yellow Pool), CP (Clear Pool), and TF (Thin Film) are all CCC_coarse_, dominated by calcite. The local bedrock (BR) also has a calcite-dominated mineralogy. In contrast, samples YS-3 and YS-4 are CCC_fine_, characterized by a mixture of calcite (Cal) and quartz (Qtz).


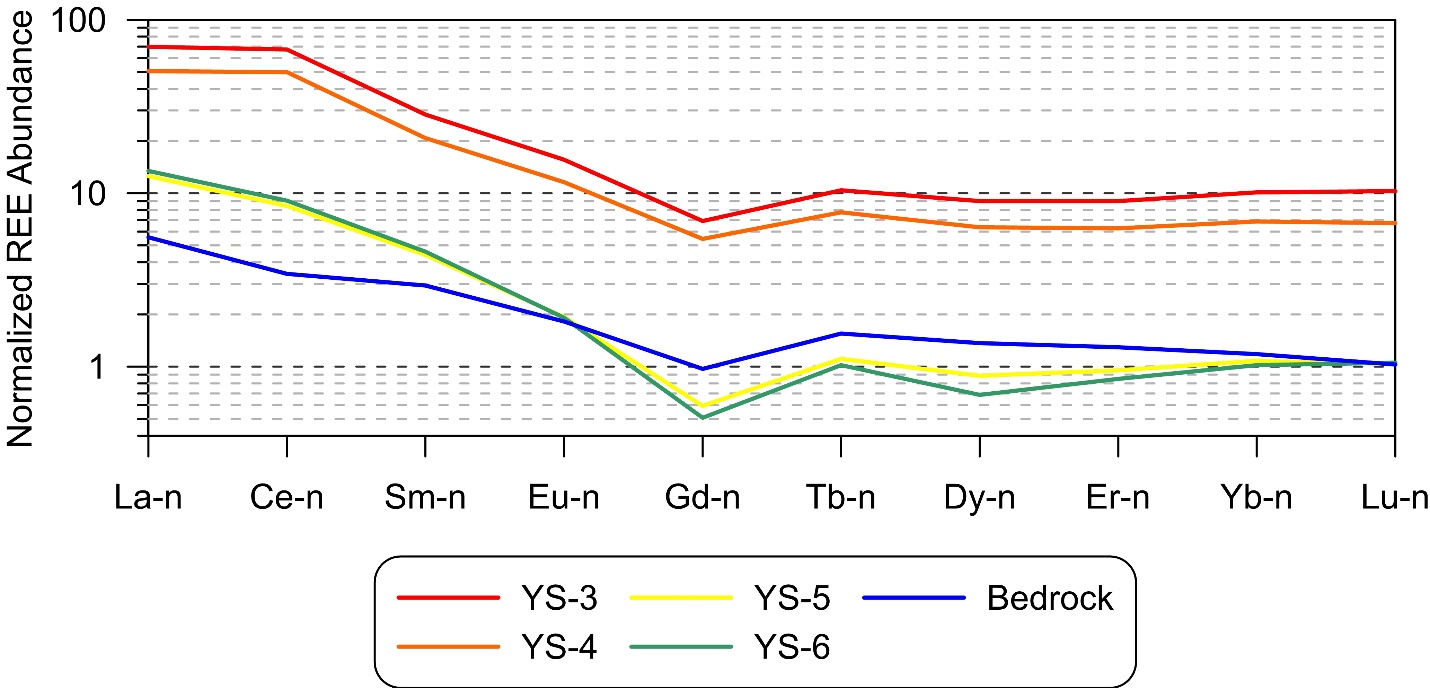


**Figure S3**: Rare earth element (REE) patterns for samples from Winter Wonderland Cave, normalized to a chondrite standard. REEs are notably more abundant in the two CCC_fine_ samples (YS-3 and YS-4), consistent with a greater detrital component.


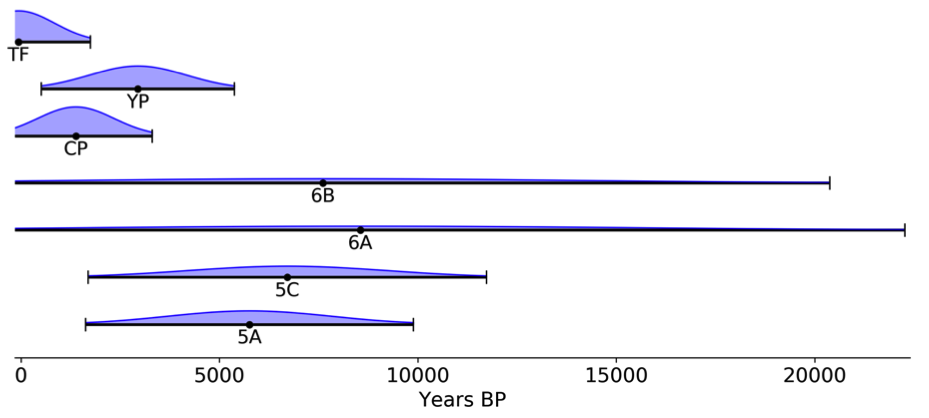


**Figure S4**. Results of U-Th dating of CCCs from Winter Wonderland Cave. The TF sample formed between 2016 and 2018, so the initial ^230^Th/^232^Th for this sample (6.89 x 10^-6^) was used for all of the samples. 5A and 5C are replicates for sample YS-5. 6A and 6B are replicates for samples YS-6. Wide error bars are a product of a large correction for detrital ^230^Th. Nonetheless, all of the dated samples likely formed during the Holocene, and ages for CP and YS-6 overlap with modern.


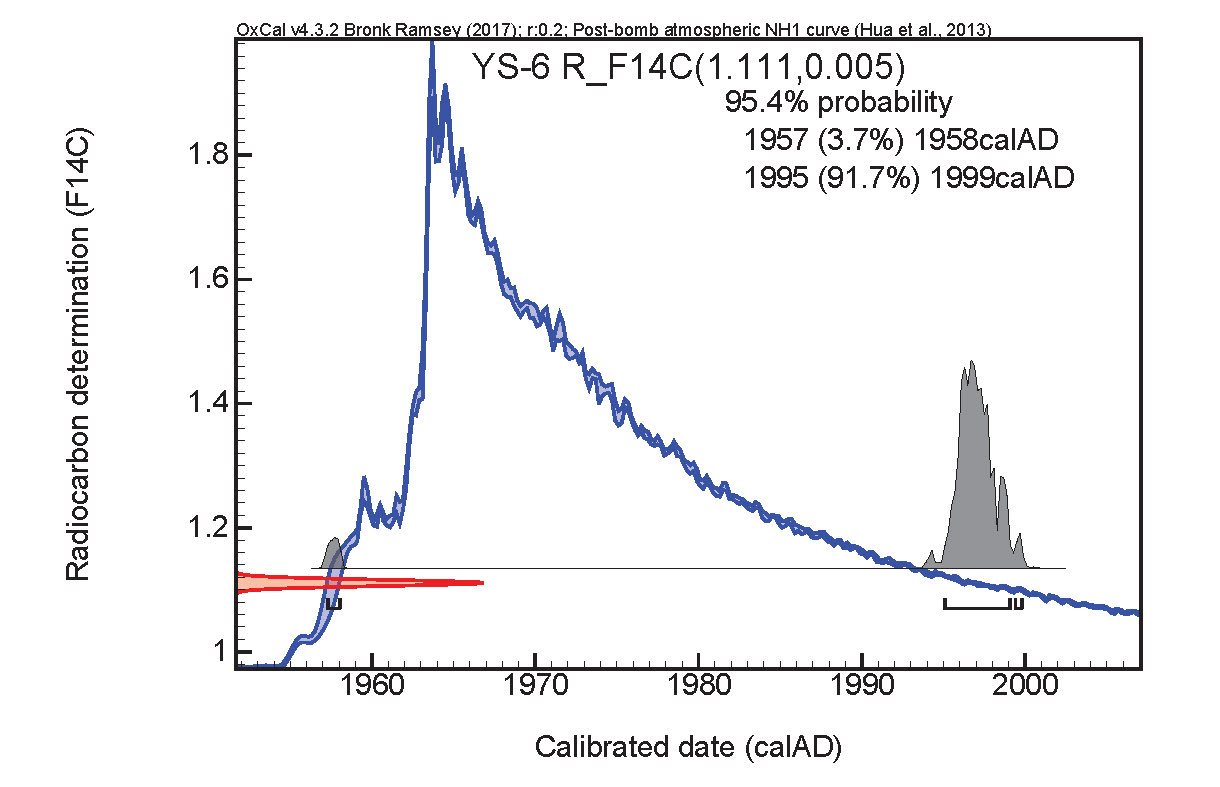


**Figure S5**. Results of radiocarbon dating of CCC_coarse_ sample YS-6. The sample contained excess ^14^C indicating formation after the peak of atmospheric bomb testing. Calibrated with the NH1 bomb curve, the sample likely formed between AD 1995 and 1999, which overlaps with the distal young end of the error range on the U-Th analysis for this sample (Fig. S4).


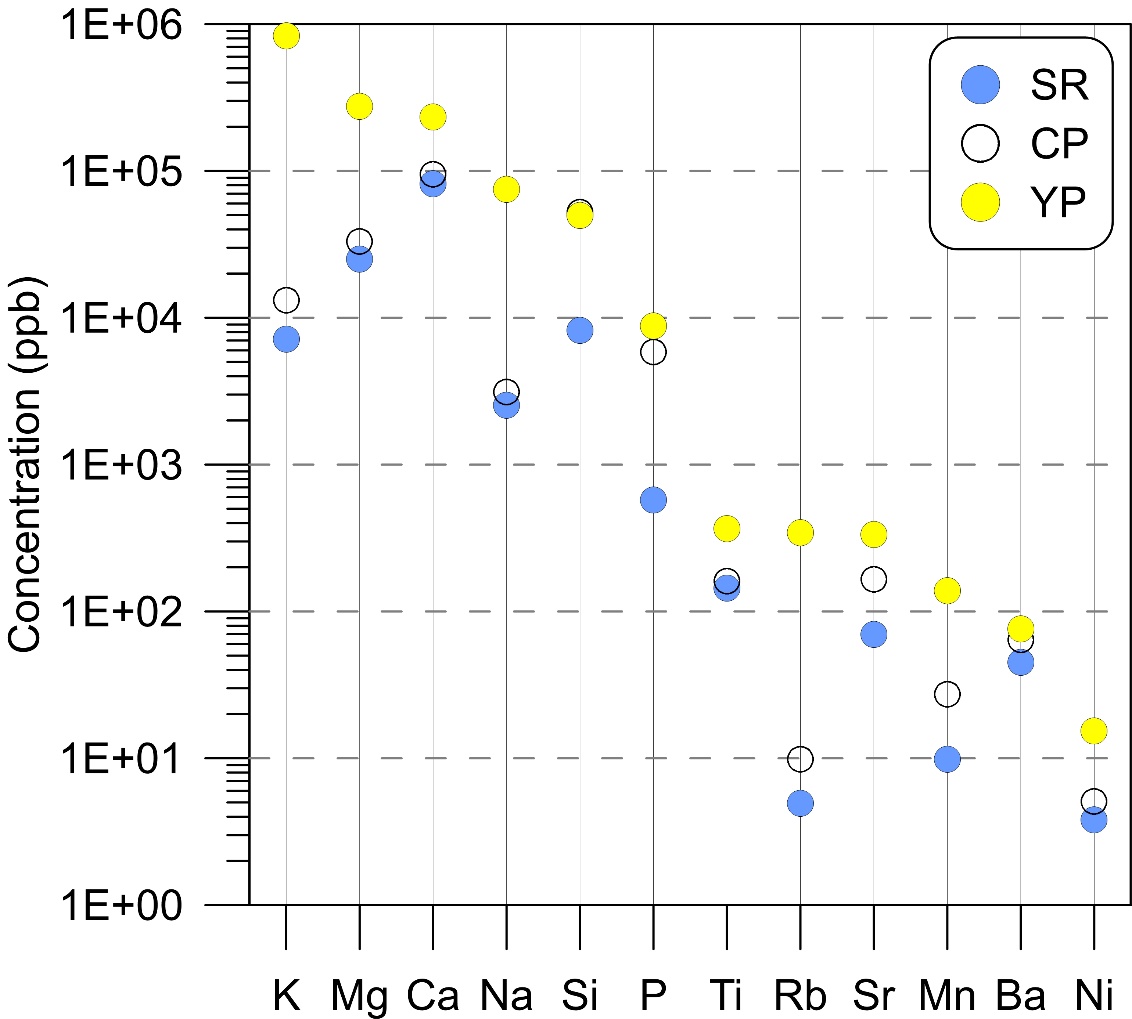


**Figure S6**. Abundance of consistently detectable elements in water samples from Winter Wonderland Cave. Sample SR was collected from a pool with a lid of ice in 2019 (Fig. 2b). Samples YP and CP were collected from pools on the ice surface in 2018 (Table S1). Concentration of all solutes is much higher in the YP (Yellow Pool) sample.
